# Dietary supplement intake in women with breast cancer before and after diagnosis: results from the SUCCESS C trial

**DOI:** 10.1186/s12885-024-12341-3

**Published:** 2024-05-15

**Authors:** Dagmar Hauner, Anna Mang, Lara Donik, Florian Schederecker, Dorothy Meyer, Brigitte Rack, Wolfgang Janni, Hans Hauner

**Affiliations:** 1 Institute of Nutritional Medicine, Else Kröner Fresenius Center for Nutritional Medicine, TUM School of Medicine and Health, Technical University of Munich, Munich, Germany; 2 Chair of Epidemiology, TUM School of Medicine and Health, Technical University of Munich, Munich, Germany; 3 Department of Gynecology and Obstetrics, University Hospital Ulm, Ulm, Germany

**Keywords:** Dietary supplement use, Inadequate intake, Excess intake, Breast cancer, Micronutrients

## Abstract

**Background:**

There is little evidence that dietary supplements are beneficial for patients with breast cancer; therefore, they are usually not recommended by treatment guidelines. The aim of the present analysis was to assess the prevalence of dietary supplement (DS) intake among women before and after a breast cancer diagnosis.

**Methods:**

Participants in the SUCCESS C lifestyle intervention study, a randomized controlled trial in women with newly diagnosed intermediate- to high-risk breast cancer, completed two questionnaires on dietary supplement intake 24 months (QS1) and 48 months (QS2) after beginning the lifestyle intervention. The study was registered on 12.17.2008 under the EU Clinical Trials Register https://www.clinicaltrialsregister.eu/, trial registration number: 2008-005453-38. The questionnaires collected data on DS intake during the 5-year period prediagnosis (QS1) and in the period postdiagnosis (QS2). Multivariate logistic regression models were fitted to examine differences in DS intake between the two intervention groups. The groups were then pooled to examine differences in DS use between the prediagnostic and postdiagnostic period.

**Results:**

A total of 320 questionnaires from 58.5% of intervention group completers and 416 questionnaires from 46.6% of low-level intervention group completers were included in the analysis. Overall, 20.2% of all respondents reported taking DS prior to their diagnosis. After a cancer diagnosis, the percentage of women taking DS significantly increased to 56.4% (p for time effect < 0.0001). No differences in DS intake between the intervention groups were observed. Single or combined preparations of vitamins and minerals/trace elements were the most frequently reported supplements. Notably, a 9-fold increase in vitamin D intake was reported postdiagnosis, where the proportion of women increased from 3.8 to 34.5%.

**Conclusion:**

A 3-fold increase in the reported intake of dietary supplements was seen in women after a breast cancer diagnosis. These observations underscore the need to incorporate patient education surrounding the use of dietary supplements in a treatment care plan, particularly addressing the negligible benefits as well as the potential risks and treatment interactions.

**Supplementary Information:**

The online version contains supplementary material available at 10.1186/s12885-024-12341-3.

## Background

Dietary supplement (DS) intake has gained popularity among patients with a wide range of diseases and can be obtained without a prescription in most countries. DS are subject to European law in the European Union [1] and complemented by national regulations. In Germany, DS are defined as food products that supplement general nutrition and consist of a “concentrate of nutrients or other substances with a nutrition-specific or physiological effect, alone or in composition” [2]. Importantly, DS do not require approval before they are marketed, and in Germany, in contrast to other countries such as Denmark and France, there is no defined upper intake level [3]. Only the amounts of the individual ingredients, per serving, must be indicated with reference to the recommended daily intake and an accompanying warning not to exceed the recommended daily amount [2]. National consumer surveys show that over one-quarter (27.6%) of adults in Germany report taking DS [4, 5].

The use of DS is also rather common among cancer patients [6, 7], and is particularly high in women with breast cancer, with reported rates ranging between 45 and 87% of patients [6, 8–14]. This behavior is in clear contrast to recommendations from expert panels, which universally discourage the use of supplements for both cancer survivors in general and breast cancer patients [15, 16]. Because of this discrepancy, we were interested in examining the prevalence of DS intake among participants in the lifestyle modification part of the German SUCCESS C trial before and after a breast cancer diagnosis.

## Methods

### The SUCCESS C trial

Data was obtained from the SUCCESS C trial, an open-label, multicenter, randomized controlled study that examined the effect of two different chemotherapy regimens (*n* = 3642) as well as the effect of a comprehensive lifestyle intervention program on disease-free survival in women with newly diagnosed HER2/neu-negative intermediate-risk to high-risk breast cancer [17–19]. The study and all experimental protocols were approved by the Heinrich Heine University Düsseldorf Ethics Committee and was registered on 12.17,2008 under the EU Clinical Trials Register https://www.clinicaltrialsregister.eu/, identifier: 2008-005453-38. All participants provided written informed consent.

**Fig. 1:**
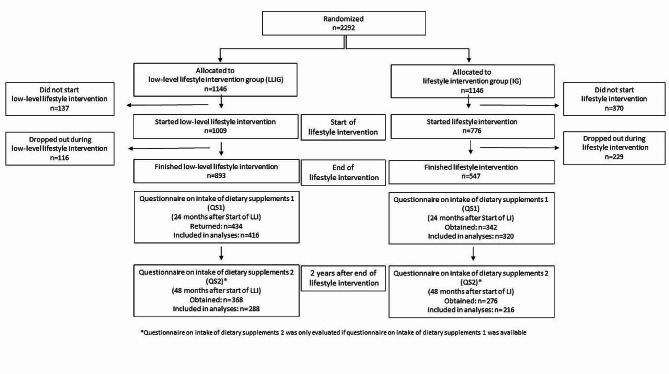
Flow diagram of the lifestyle intervention part of SUCCESS C Trial and the analysis of the intake of dietary supplements

The focus of the 2-year lifestyle intervention was on moderate weight loss in breast cancer patients with a BMI between 24 and 40 kg/m² (*n* = 2292). Not later than six weeks after surgery, 2292 women were randomized to either the lifestyle intervention group (IG) or the low-level intervention group (LLIG). The lifestyle intervention started either 3 or 6 months after the completion of chemotherapy. Women in the IG received an individualized, telephone-based program promoting an energy-reduced, healthy diet and regular physical activity, whereas the LLIG received general recommendations for a healthy lifestyle [18]. Figure 1 shows a participation flowchart. From the 776 women (IG) and 1009 women (LLIG) who began the lifestyle program, at total of 547 in the IG (70.5%) and 893 in the LLIG (88.5%) completed the 2-year intervention. Specific information or advice on dietary supplements was not delivered to either group.

### Assessment of dietary supplement intake

Participants in both groups were requested to record their food intake, using 7-day dietary records, as well as their physical activity. These data were collected at five time points (i.e., baseline, 6, 12, 24 and 48 months after starting the lifestyle intervention). All participants were also requested to fill in two additional questionnaires on DS intake, developed by our research team and added to the dietary record forms, at 24 months (i.e. the end of the lifestyle intervention, QS1) and 48 months (i.e. 24 months later, QS2). The questionnaires collected data on DS intake for the following periods: (1) within 5 years prior to a breast cancer diagnosis (T0), and (2) postdiagnosis at 24 months (T1) and 48 months (T2) after starting the lifestyle intervention (Table S1).

Participants were asked whether they used DS, and if so, to report the dosage, frequency (i.e. how often in a day), and product name of any vitamins, minerals/trace elements, and/or any combinations of vitamins and minerals and/or any other dietary supplements.

### Statistical methods

This secondary analysis used data from 736 women who completed questionnaires on DS intake. Descriptive data were derived from QS1 and reported as mean values ± standard deviation (SD).

The components and dosages of DS were calculated from questionnaires, when available. Any drugs that were misclassified as DS by participants were excluded from analysis.

Analyses were performed with IBM SPSS Statistics version 26 (IBM, Armonk, NJ, USA) and R software version 4.0.3 (RStudio Inc, Boston MA, USA). The proportion of women taking DS were compared between the two intervention groups using binary logistic regression models. The primary aim of this analysis was to investigate any differences in DS prevalence before (T0) and after a breast cancer diagnosis (T1) for the entire cohort by fitting binary logistic regression models. We further investigated the change in DS intake in the period postdiagnosis (i.e. the change between T1 and T2). Only women who filled out questionnaires at both time points were included in the descriptive data presentation.

Subgroups were determined *a priori* and analyses were performed to compare the following groups: participants with a BMI < 30 kg/m² vs. BMI ≥ 30 kg/m², and participants with positive vs. negative hormone receptor status. Participants were also subcategorized into three age groups: < 51 years, 51–64 years and ≥ 65 years to observe any differences in DS use. The SUCCESS C study was not powered to detect differences between subgroups; hence mainly descriptive reporting of between-group differences was considered the most appropriate choice rather than using interaction tests to assess subgroup effects. This approach was chosen to reduce the risk of Type 1 errors [20]. All models were adjusted for age at baseline, weight at baseline and chemotherapy arm. A two-tailed p value of ≤ 0.05 was considered statistically significant.

## Results

In total, 342 women in the IG and 434 women in the LLIG filled out the QS1, of which 320 (from 58.5% of the IG completers) and 416 (46.6% of the LLIG completers) could be included in the analyses. For QS2, we obtained 276 (IG) and 368 (LLIG) questionnaires, of which 216 (IG) and 288 (LLIG) provided complete questionnaires at both time points to assess the change in DS use over time (Fig. 1). The discrepancy in the number of collected questionnaires versus those included in the analyses was due to several questionnaires containing either insufficient or implausible responses. Comparing women whose QS1 were missing or incomplete (*n* = 712) to those who were included in this study (*n* = 736), we found that the non-included women were younger (55.5 vs. 57.4 years), had a higher BMI (29.4 vs. 28.6 kg/m^2^) and an increased waist circumference (94.6 vs. 92.9 cm). Moreover, a larger proportion of these women were premenopausal (37.4% vs. 27.6%), smokers (17.1% vs. 11.0%) and underwent an anthracyclin-based chemotherapy regimen (52.5% vs. 47.0%) (all p-values < 0.05).

The baseline participant characteristics on dietary supplement intake are presented in Table 1 and stratified by intervention group. Findings were generally comparable in both groups. The mean age was 57.4 years (both groups) and mean BMI was 28.8 kg/m² (IG) and 28.5 kg/m² (LLIG). Baseline weight was comparable in both groups (IG 78.1 kg and LLIG 77.3 kg). Most participants had tumor staging T1 or T2, N0 or N1 status, and G2 or G3 grading. The majority of women were hormone receptor positive (IG 83.7% and LLIG 79.3%). Most women, 77.2% (IG) and 79.6% (LLIG), underwent breast-conserving surgery, whereas 18.8% (IG) and 17.5% (LLIG) had mastectomies. All participants received adjuvant chemotherapy and most underwent radiation therapy (IG 90.9% and LLIG 89.9%). Hypertension was noted in 40.0% (IG) and 39.2% (LLIG) of the participants, diabetes mellitus in 4.7% (IG) and 4.8% (LLIG), and coronary heart disease in 0% (IG) and 1.0% (LLIG) (Table 1).

### Total intake and intake of specific groups of dietary supplements

Prediagnostic DS use was reported by 20.2% and postdiagnostic use by 56.4% of all women. Results were similar when groups were split into IG and LLIG, with no significant between-group differences at either time point (Table 2). After a breast cancer diagnosis, a significant 2.8-fold increase (p-value for time effect < 0.0001) in DS use was found in all participants, which was also similar when observing the behavior of each group separately (2.6-fold in IG and 2.9-fold in LLIG, with no significant group difference) (Table 2).

We found that 13.9% of women reported taking any mineral/trace mineral, 11.1% any vitamin, and 8.3% any combination of vitamins and/or minerals before diagnosis. Postdiagnosis, the use of DS increased significantly by 4.1-fold for any vitamin (45.9% of women), 3.2-fold for any mineral/trace element (44.7% of women), and 3.9-fold for any combination of vitamins and/or minerals (31.9% of women) (p for time effect < 0.0001 for all three). Similar results were found for the IG and the LLIG separately, with no significant between-group differences for either time point. The consumption of herbal supplements (T0 4.5% vs. T1 11.8%), omega-3 fatty acid supplements (T0 1.5% vs. T1 2.9%) and other supplements (T0 1.6% vs. T1 8.0%) was less frequently reported (Table 2).

### Intake of specific micronutrients

We found that prediagnosis, women most frequently took magnesium (9.9%), followed by zinc (5.4%), vitamin C (5.2%), B vitamins (4.6%) and vitamin D (3.8%). Fewer women reported taking vitamin E (2.2%), calcium (2.3%), selenium (1.6%), beta-carotene (1.2%) and vitamin A (1.1%). After a breast cancer diagnosis, 1 out of 3 women (34.5%) reported taking vitamin D. A considerable increase in intake was also reported for B vitamins (21.6%), magnesium (21.2%), zinc (19.6%), selenium (17.4%), vitamin C (13.6%) and calcium (12.8%). Fewer women reported taking vitamin E (8.8%), vitamin A (6.5%), and beta-carotene (5.2%). The increase in DS use postdiagnosis was particularly notable for selenium, vitamin D and calcium (p for time effect < 0.0001). No significant between-group differences were found when comparing DS use in the IG vs. LLIG (Table 3).

### Dosage of specific micronutrients

The dosage of selected micronutrients that were taken postdiagnosis is shown in Table S2. The median daily dosage values were 310.0 mg/d for vitamin C, 22.0 µg/d for vitamin D, 38.0 mg/d for vitamin E, 600.0 mg/d for calcium, 300.0 mg/d for magnesium, 100.0 mg/d for selenium, and 10.0 mg/d for zinc. A high percentage of women who reported using selected micronutrients took dosages that exceeded the reference values for daily intake (Table S3), such as vitamin C (85.5%), vitamin E (80.0%), zinc (78.6%), selenium (66.4%), and vitamin D (50.7%), followed by magnesium (31.3%), and calcium (14.6%) (Table S2). Women taking dosages exceeding the tolerable upper intake level (UL) (Table S3) were reported for magnesium (53.1%), zinc (20.0%) vitamin E (13.3%), selenium (8.4%), vitamin C (4.4%), vitamin D (3.0%), and calcium (2.3%) (Table S2).

### Subgroup analyses

A similar significant increase in the intake of total DS was found for women with a BMI < 30 kg/m² and women with a BMI ≥ 30 kg/m² (p for time effect < 0.0001) as well as for women with and without anti-hormonal therapy (data not shown). Similar increases in postdiagnosis DS use were seen when participants were classified into three age-range categories (T0 vs. T1 in women < 51 years old: from 22.1 to 58.4%; 51–64 years old: from 19.2 to 56.4% and in women ≥ 65 years old: from 20.3 to 54.2%).

### Temporal changes of postdiagnosis DS intake

No significant differences were observed when analysis was restricted to participants who filled out both QS1 and QS2 at the end of active lifestyle intervention (T1) and at a follow-up two years later (T2). Overall, DS intake was reported by 58.3% (*n* = 294) of women at T1 and 57.9% (*n* = 292) at T2; 48.0% reported taking any vitamin supplement at T1 and 46.6% at T2, 46.6% took any mineral/trace mineral at T1 and 43.1% at T2 and 42.3% took any combination of vitamins/minerals and/or other dietary supplements at T1 and 41.5% at T2 (Table S4).

## Discussion

The main finding of this secondary analysis was that women increased their use of DS almost 3-fold, from 20.2 to 56.4%, after receiving a breast cancer diagnosis. The proportion of participants taking DS was similar when the cohort was split into their respective study intervention groups (i.e., IG and LLIG). Confounders, such as age, BMI or type of cancer treatment did not substantively change the outcomes.

Data from a nationally representative sample showed that 28.0% of women in Germany report taking DS, with even higher rates observed among older women (e.g. 43.2% of women aged 65–80 years) [4]. Our findings show that the percentage of SUCCESS C participants who took DS was clearly higher than those who were part of the national sample, irrespective of age. The prevalence we report is comparable to findings from a recent German survey that found that 59.8% of women with breast cancer took DS after their diagnosis [14]. Notably, another German study in women with breast cancer observed that almost twice as many women compared to our cohort (36%) took DS prediagnosis and observed only a moderate increase in prevalence after diagnosis (45%) [13].

Our observations of a postdiagnostic increase in DS intake is in line with other cross-sectional and prospective studies. European studies have reported postdiagnostic DS intake rates of 62.8% in women with cancer [10] and 68.3% in individuals with breast cancer [21]. It is noteworthy that US-American studies show higher prevalence rates in both the prediagnostic (i.e. 54 − 84%) [9, 10, 12] and postdiagnostic periods (i.e. 60.6% and 87.0%) [6, 7, 9, 11, 12, 22], reflecting a generally higher DS intake compared to European studies. Of interest, some of these studies showed that adult cancer survivors with a healthier lifestyle, lower BMI, higher diet quality, and higher physical activity were more likely to use DS [7, 21, 23].

Single or combined preparations of vitamins and minerals/trace minerals were the most frequently reported DS in the SUCCESS-C trial, with a significant 3- to 4-fold increase postdiagnosis, a finding that is in line with data from another European study [10]. The widespread use of DS, reported in these studies, is in striking contrast to current recommendations for breast cancer survivors. The Continuous Update Expert Report from 2018 [15] as well as national expert statements [24] explicitly do not recommend the use of DS in this population. In addition, the US Preventive Services Task Force recently released an updated report on the evidence for the efficacy and safety of DS for the prevention of cancer and cardiovascular diseases. In this report, a clear recommendation against beta carotene and vitamin E supplements for cancer patients was given [16]. The authors also noted that only insufficient evidence was found to evaluate the benefits and potential harms of multivitamins, vitamins B3, B6, C and D, calcium, selenium, and folic acid (both with and without vitamin B12) on cancer outcomes [16]. In accordance with this report, literature confirms that there is not only no proven benefit for DS, but also the potential for negative effects which must be considered when taking any DS [16, 24–31].

Given compelling evidence indicating the absence of benefits from dietary supplements, their widespread use by a majority of breast cancer patients is concerning. One reason for this discrepancy is the inadequate translation of established scientific knowledge and research findings into clinical practice to inform patient care and improve health literacy. In the SUCCESS C trial, no specific information on the use of DS was delivered to either group. Therefore, the marked increase in DS intake following a cancer diagnosis, found in this analysis, likely did not result from medical recommendations provided by their oncologists. A large breast cancer trial observed that one-third of participants were advised to take DS by their clinicians and 10% were asked to discontinue DS intake, while 51% of the patients did not receive any advice [32]. These findings suggest that the widespread use of DS is not medically supervised. Other research indicates that the majority of physicians are unaware of DS use among their cancer patients [6]. A web-based survey conducted as part of the NutriNet Sante Cohort study noted potentially adverse DS intake patterns among a significant number of cancer patients. Examples include concurrently taking vitamin E and anticoagulants or smokers taking beta-carotene supplements [21].

The general lack of awareness among clinicians on their cancer patients’ use of DS aligns with a growing trend toward self-medication and a heightened interest in complementary and alternative medicine [33]. At the same time, predatory marketing campaigns targeting cancer patients and offering deceptive or unsupported health claims are common. Patients are vulnerable to such messages and frequently believe that DS can prevent nutritional deficiencies and thereby support their immune system. Furthermore, many patients hope or expect that DS can mitigate the side effects of conventional cancer therapies and improve their quality of life [34].

Vitamin D supplementation showed the most dramatic increase postdiagnosis in the present study: from 3.8 to 34.5% of participants. Similarly, in a French study, vitamin D was clearly preferred among all single-ingredient DS, where 47.7% of women with breast cancer reported regular intake [21]. The reasons for these high intake rates are unclear. According to current recommendations by the World Cancer Research Fund there is insufficient evidence for general vitamin D use for this population [15, 24], although some guidelines recommend routine measurement of serum 25(OH)D in oncology patients and supplementation if a deficiency is detected [35]. For osteoporosis prevention in postmenopausal women, consuming the recommended daily amounts of calcium and vitamin D should be ensured through a balanced diet. If the recommended levels cannot be achieved through food then supplements should be given to fill nutrient gaps [36]. Vitamin D supplements are principally recommended when clinical osteoporosis is diagnosed [36]. In breast cancer patients, prevention of therapy-associated bone loss is advised. However, vitamin D and calcium supplementation should be medically indicated and tailored to the individual patient [24]. Therefore, the increase in vitamin D supplementation we observed in this cohort is plausible and at least partially justified.

A high-quality diet is associated with a better breast cancer prognosis [37] and routine monitoring for nutritional imbalances and weight change is important for all cancer patients [38, 39]. During chemotherapy and radiotherapy, an adequate intake of micronutrients should be ensured according to physiological requirements [24]. However, possible drug interactions must be considered. While some antioxidants might reduce side effects, antagonistic effects of antioxidants and other nutrients may compromise the therapeutic efficiency of chemotherapy and radiotherapy, thereby affecting prognostic outcomes [24, 25, 27, 30, 40, 41]. Hence, unnecessary and unmonitored consumption of dietary supplements should be avoided, particularly in excessive doses.

Finally, there is also a financial burden on patients arising from out-of-pocket costs for DS that cannot be ignored. Many cancer patients suffer from financial hardship as a consequence of their illness due to diverse additional costs [42], a reduction in working hours [43], a loss of revenue and an increased likelihood of unemployment [43]. Around 20–30% of cancer survivors do not return to the workplace [42]. Financial challenges could potentially exacerbate the disease burden and hinder the adoption of a healthy lifestyle. For these reasons, clinicians bear the responsibility of addressing costs associated with DS use within the patient treatment plan to prevent avoidable additional expenses [15, 39].

Our manuscript presents several strengths that contribute to the robustness of our findings. Firstly, our study was able to analyze data on DS intake from a sizable cohort of 736 participants. Additionally, we collected data from DS use in both the pre- and postdiagnostic periods, providing a nuanced understanding of changes in DS intake behavior. Furthermore, detailed baseline characteristics of participating women were documented, enhancing the reliability and applicability of our results. The nationwide recruitment of women from over 200 gynecological practices across Germany underscores the potential generalizability of our findings to adult German women with overweight or obesity and Her2/neu-negative breast cancer.

However, several weaknesses also merit consideration. Notably, data on DS use was available from only around one-third of the initially randomized participants and around 40% of those who commenced the lifestyle intervention. This may have resulted in an overall discrepancy between the DS use we report in this study and behavior from the entire cohort, although other studies have reported similar findings. Additionally, we lacked information regarding the reasons for DS intake among participants and relied solely on retrospective self-reporting. Lastly, the study was limited to women with a BMI between 24 and 40 kg/m² and Her2/neu-negative breast cancer who received chemotherapy, potentially restricting the generalizability of our findings to a broader population. These weaknesses highlight areas for further investigation and consideration in future research endeavors.

In conclusion, results from this study show that the proportion of women taking DS markedly increased after a diagnosis of breast cancer. Given the inadequate scientific evidence supporting the general benefit and safety of additional DS intake, patients with breast cancer should receive fundamental guidance from their treating physicians regarding DS. Consideration of DS use should be a recurring and consistently addressed aspect of comprehensive cancer care.

**Table 1:** Baseline characteristics of the participants included in the analyses of dietary supplement intake

Participant characteristics	Low-level intervention group	Intervention group
Number of participants, n (%)	416 (56.5)	320 (43.5)
**A. General characteristics**		
Age, years (± SD)	57.4 (± 9.29)	57.4 (± 8.76)
Height, cm (± SD)	164.6 (± 6.09)	164.5 (± 6.33)
Weight, kg (± SD)	77.3 (± 11.30)	78.1 (± 11.61)
Body Mass Index, kg/m^2^ (± SD)	28.5 (± 3.80)	28.8 (± 3.53)
Body Mass Index Category, n (%)		
BMI < 30.0 kg/m^2^	273 (66.1)	219 (68.4)
BMI ≥ 30.0 kg/m^2^	140 (33.9)	101 (31.6)
Menopause status, n (%)		
Postmenopausal	310 (74.5)	223 (69.7)
Premenopausal	106 (25.5)	97 (30.3)
Smoking, n (%)		
No	324 (86.6)	267 (92.1)
Yes	50 (13.4)	23 (7.9)
**B. Tumor characteristics**, n (%)		
Tumor status		
pT1	184 (44.2)	146 (45.6)
pT2	203 (48.8)	150 (46.9)
pT3	19 (4.6)	21 (6.6)
pT4	10 (2.4)	3 (0.9)
Nodal status		
0	167 (40.1)	110 (34.4)
1	205 (49.3)	172 (53.8)
2	36 (8.7)	27 (8.4)
3	8 (1.9)	11 (3.4)
Grading		
G1 (well differentiated)	29 (7.0)	26 (8.1)
G2 (moderately differentiated)	229 (55.0)	169 (52.8)
G3 (poorly differentiated)	158 (38.0)	125 (39.1)
Hormonal receptor status		
Negative	86 (20.7)	52 (16.3)
Positive	330 (79.3)	268 (83.7)
Estrogen receptor		
ER-	90 (21.6)	55 (17.2)
ER+	326 (78.4)	265 (82.8)
Progesterone receptor		
PR-	123 (29.6)	76 (23.8)
PR+	293 (70.4)	244 (76.3)
**C. Treatment**, n (%)		
Radiation therapy		
No	42 (10.1)	29 (9.1)
Yes	374 (89.9)	291 (90.9)
Surgery type		
Breast conserving	331 (79.6)	247 (77.2)
Mastectomy	73 (17.5)	60 (18.8)
Others	12 (2.9)	13 (4.1)
Chemotherapy arm		
A	189 (45.4)	157 (49.1)
B	227 (54.6)	163 (50.9)
**D. Cardiometabolic diseases**,n (%)		
Diabetes mellitus		
No	396 (95.2)	305 (95.3)
Yes	20 (4.8)	15 (4.7)
Hypertension		
No	252 (60.6)	191 (59.7)
Yes	163 (39.2)	128 (40.0)
Coronary heart disease		
No	412 (99.0)	320 (100.0)
Yes	4 (1.0)	0 (0.0)

Data are mean (SD) or n (%);

SD, standard deviation.

**Table 2:** Intake of dietary supplements before and after a breast cancer diagnosis

Time	LLIG *n* (%) (*n* = 416)	IG *n* (%) (*n* = 320)	Total *n* (%) (*n* = 736)	OR, 95% CI	Between group difference ^a^	Time effect until T1^b^
**Total intake of dietary supplements**						
**T0**	80 (19.2)	69 (21.6)	149 (20.2)	1.02 (0.97–1.09)	0.420	-
**T1**	233 (56.0)	182 (56.9)	415 (56.4)	1.01 (0.94–1.09)	0.749	< 0.0001
**Any vitamin** ^**c**^						
T0	41 (9.9)	41 (12.8)	82 (11.1)	1.03 (0.99–1.08)	0.188	-
T1	187 (45.0)	151 (47.2)	338 (45.9)	1.03 (0.95–1.10)	0.483	< 0.0001
**Any mineral/trace mineral** ^**c**^						
T0	53 (12.7)	49 (15.3)	102 (13.9)	1.03 (0.98–1.08)	0.230	-
T1	184 (44.2)	145 (45.3)	329 (44.7)	1.01 (0.94–1.09)	0.710	< 0.0001
**Any combination of vitamins and/or minerals** ^c, d^						
T0	32 (7.7)	29 (9.1)	61 (8.3)	-	-	-
T1	129 (31.0)	106 (33.1)	235 (31.9)	1.02 (0.95–1.09)	0.546	< 0.0001
**Any product with herbal ingredients**, ^c, e^						
T0	17 (4.1)	16 (5.0)	33 (4.5)	-	-	-
T1	50 (12.0)	37 (11.6)	87 (11.8)	-	-	-
**Any product with omega-3 fatty acids** ^**c**^						
T0	5 (1.2)	6 (1.9)	11 (1.5)	-	-	-
T1	9 (2.2)	12 (3.8)	21 (2.9)	-	-	-
**Other products** ^**c**, f^						
T0	5 (1.2)	7 (2.2)	12 (1.6)	-	-	-
T1	33 (7.9)	26 (8.1)	59 (8.0)	-	-	-

^a^ Between group difference in intake of dietary supplements was tested using binary logistic regression, adjusted for age at baseline, weight at baseline and chemotherapy arm

^b^ Differences in dietary supplement intake from T0 to T1 for the total cohort was tested using binary logistic regression, adjusted for age at baseline, weight at baseline and chemotherapy arm

^c^ In monopreparation or in combination

^e^ Products with herbal ingredients, e.g.: Artichoke, cranberry, curcuma, gingko, mistletoe, mushrooms, pineapple, spirulina, valerian

^f^ Other supplements, e.g.: Bacteria, coenzyme Q10, collagen, glucosamine, hyaluronic acid, silica.

OR not calculated for *n* < 50 (LLIG + IG)

CI, confidence interval; IG, intervention group; LLIG, low-level intervention group; n, number of participants with included QS; OR, odds ratio; T0, dietary supplement intake before diagnosis of breast cancer; T1, dietary supplement intake after diagnosis of breast cancer until the end of the lifestyle intervention

**Table 3:** Intake of specific micronutrients of dietary supplements before (T0) and after (T1) a breast cancer diagnosis

Time	LLIG *n* (%) (*n* = 416)	IG *n* (%) (*n* = 320)	Total *n* (%) (*n* = 736)	OR, 95% CI	Between group difference ^a^	Time effect until T1 ^b^
**Vitamin A**						
T0	3 (0.7)	5 (1.6)	8 (1.1)	-	-	-
T1	32 (7.7)	16 (5.0)	48 (6.5)	-	-	
**Beta carotene**						
T0	4 (1.0)	5 (1.6)	9 (1.2)	-	-	-
T1	22 (5.3)	16 (5.0)	38 (5.2)	-	-	
**B vitamins**						
T0	21 (5.0)	13 (4.1)	34 (4.6)	-	-	-
T1	94 (22.6)	65 (20.3)	159 (21.6)	0.98 (0.92–1.04)	0.445	< 0.0001
**Vitamin C**						
T0	19 (4.6)	19 (5.9)	38 (5.2)	-	-	-
T1	57 (13.7)	43 (13.4)	100 (13.6)	1.0 (0.95–1.05)	0.848	< 0.0001
**Vitamin D**						
T0	15 (3.6)	13 (4.1)	28 (3.8)	-	-	-
T1	142 (34.1)	112 (35.0)	254 (34.5)	1.01 (0.94–1.08)	0.749	< 0.0001
**Vitamin E**						
T0	10 (2.4)	6 (1.9)	16 (2.2)	-	-	-
T1	42 (10.1)	23 (7.2)	65 (8.8)	0.97 (0.93–1.01)	0.176	< 0.0001
**Calcium**						
T0	12 (2.9)	5 (1.6)	17 (2.3)	-	-	-
T1	53 (12.7)	41 (12.8)	94 (12.8)	1.00 (0.95–1.05)	0.946	< 0.0001
**Magnesium**						
T0	42 (10.1)	31 (9.7)	73 (9.9)	1.00 (0.95–1.04)	0.873	
T1	94 (22.6)	62 (19.4)	156 (21.2)	0.97 (0.91–1.02)	0.248	< 0.0001
**Selenium**						
T0	6 (1.4)	6 (1.9)	12 (1.6)	-	-	-
T1	71 (17.1)	57 (17.8)	128 (17.4)	1.01 (0.95–1.07)	0.755	< 0.0001
**Zinc**						
T0	18 (4.3)	22 (6.9)	40 (5.4)	-	-	-
T1	85 (20.4)	59 (18.4)	144 (19.6)	0.98 (0.93–1.04)	0.545	< 0.0001

^a^ Between group differences in dietary supplement intake was tested using binary logistic regression, adjusted for age at baseline, weight at baseline and chemotherapy arm

^b^ Difference in dietary supplement intake from T0 to T1 for the total cohort was tested using binary logistic regression, adjusted for age at baseline, weight at baseline and chemotherapy arm

OR not calculated for *n* < 50 (LLIG + IG)

CI, confidence interval; IG, intervention group; LLIG, low-level intervention group; n, number of participants with included QS; OR, odds ratio; T0, dietary supplement intake before diagnosis of breast cancer; T1, dietary supplement intake after diagnosis of breast cancer until end of lifestyle intervention

### Electronic supplementary material

Below is the link to the electronic supplementary material.

Supplementary Material 1

## Data Availability

The datasets used and/or analyzed during the current study are available from the corresponding author on reasonable request.

## Abbreviations

DS: Dietary supplements
IG: Intervention group
LLIG: Low-level intervention group
QS: Dietary supplement questionnaire
