# Supplementary material for: Dietary supplement intake in women with breast cancer before and after diagnosis: results from the SUCCESS C trial
Source: BMC Cancer. 2024 May 15;24:591. doi: 10.1186/s12885-024-12341-3 (PMC11094984; doi:10.1186/s12885-024-12341-3)
Supplement: Supplementary file 1 — Supplementary Material 1 [file 12885_2024_12341_MOESM1_ESM.pdf]

Dietary supplement intake in women with breast cancer before and after diagnosis:
Results from the SUCCESS C Trial

SUPPLEMENTARY MATERIAL

Hauner D[1], Mang A[1], Donik L[1], Schederecker F[1,2], Meyer D[1], Rack B[3], Janni W[3], Hauner H[1]

[1] Institute of Nutritional Medicine, Else Kröner Fresenius Center for Nutritional Medicine, TUM School of Medicine and Health, Technical University of Munich, Munich, Germany
[2] Chair of Epidemiology, TUM School of Medicine and Health, Technical University of Munich, Munich, Germany
[3] Department of Gynecology and Obstetrics, University Hospital Ulm, Ulm, Germany

Address for correspondence:
Hans Hauner, MD
Institute for Nutritional Medicine, Else Kröner Fresenius Center for Nutritional Medicine, TUM School of Medicine and Health, Technical University of Munich, Georg-Brauchle-Ring 62, 80992 Munich, Germany

Supplementary Table 1: Dietary Supplement Survey

SUCCESS[C] Dietary Supplement Survey

D.O.B: Today's date:
Patient ID number:

Question 1) Have you taken any non-prescription dietary supplements since your breast cancer diagnosis?

Vitamins (e.g. Vitamins A, C, D, E) yes ☐ no ☐ I'm not sure ☐

List of the vitamin(s) taken (Name of product)	How often? (i.e. how many tablets, capsules, or other doses per day)	Over what time period? (start year – end year)

Minerals and/or micronutrients? (e.g. Selenium, Zinc, etc.) yes ☐ no ☐ I'm not sure ☐

List of minerals or micronutrients taken (Name of the product)	How often? (i.e. how many tablets, capsules, or other doses per day)	Over what time period? (start year – end year)

Any other supplements or combination supplements? (e.g. fish oil) yes ☐ no ☐ I'm not sure ☐

List of supplements or combination supplements taken (Name of the product)	How often? (i.e. how many tablets, capsules, or other doses per day)	Over what time period? (start year – end year)

Question 2) Did you take any dietary supplements in the last 5 years before your breast cancer diagnosis? (Please be as detailed as possible)

List of dietary supplements (Name of the product)	How often? (i.e. how many tablets, capsules, or other doses per day)	Over what time period? (start year – end year)

Supplementary Table 2: Dosages of the specific micronutrients per day: Comparison with the reference values and the tolerable upper intake levels

Time	N [a]	N [b]	Median (Min, Max) (related to N [a])	> Reference value for nutrient intake (D-A-CH) n [c] (%) (related to N [a])	> UL (EFSA [e] NA [f]) n [d] (%) (related to N[a])
**Vitamin C (mg/d)**					
T0	17	38	240.0 (60.0, 3600.0)	-	-
T1	69	100	310.0 (11.1, 19000.0)	59 (85.5)	3 (4.4) [f]
**Vitamin D (µg/d)**					
T0	7	28	10.0 (10.0, 20.0)	-	-
T1	134	254	22.0 (1.5, 500.0)	68 (50.7)	4 (3.0) [e]
**Vitamin E (mg/d)**					
T0	13	16	30.0 (6.0, 560.0)	-	-
T1	60	65	38.0 (3.0, 3000.0)	48 (80.0)	8 (13.3) [e]
**Calcium (mg/d)**					
T0	16	17	400.0 (54.0, 1000.0)	-	-
T1	89	94	600.0 (120.0, 3000.0)	13 (14.6)	2 (2.3) [e]
**Magnesium (mg/d)**					
T0	17	73	300.0 (60.0, 1080.0)	-	-
T1	64	156	300.0 (50.0, 1200.0)	20 (31.3)	34 (53.1) [e]
**Selenium (µg/d)**					
T0	11	12	55.0 (20.0, 200.0)	-	-
T1	119	128	100.0 (10.0, 1000.0)	79 (66.4)	10 (8.4) [e]
**Zinc (mg/d)**					
T0	13	40	15.0 (5.0, 25.0)	-	-
T1	70	144	10.0 (5.0, 200.0)	55 (78.6)	14 (20.0) [e]

[a] Number of reported dosage data of the specific micronutrient.
[b] Number of total reported intake of the specific micronutrient.
[c] Number of patients exceeding the reference value of the daily recommended intake according to D-A-CH (1)
[d] Number of patients exceeding the UL.
[e] Number of patients exceeding the UL according to the European Food Safety Authority (EFSA).(2)
[f] Number of patients exceeding the UL according to the National Academies (3).
No calculation for N < 50
D, days; µg, micrograms; mg, milligrams; EFSA, European Food Safety Authority; NA, National Academies; T0, intake of dietary supplements before diagnosis of breast cancer; T1, intake of dietary supplements after diagnosis of breast cancer until end of lifestyle intervention; UL, tolerable upper intake level.

Supplementary Table 3: Reference values of the daily recommended micronutrient intake and of the tolerable upper intake level for women

	Vitamin C (mg/d)	Vitamin E (mg/d)	Vitamin D (µg/d)	Selenium (µg/d)	Zinc (mg/d)	Calcium (mg/d)	Magnesium (mg/d)
**Reference value for nutrient intake (D-A-CH [a], > 50 y)**	**95**	**12 [d]**<br>**11 [e]**	**20**	**60**	**8 [f]**	**1000**	**300**
**UL (EFSA [b], adults)**	No adequate data to derive a UL	**300**	**100**	**300**	**25**	**2500**	**250**
**UL (NA [c], > 50 y)**	**2000**	1000	100	400	40	2000	350

[a] Reference values for nutrient intake according to D-A-CH (1)
[b] Reference values for nutrient intake according to the European Food Safety Authority (EFSA) (2)
[c] Reference values for nutrient intake according to the National Academies (3)
[d] For 51 - 64 y.
[e] For ≥ 65 y.
[f] For mean phytate intake.
D, days; µg, micrograms; mg, milligrams; y, years; UL, tolerable upper intake level.

Supplementary table 4: Intake of dietary supplements after diagnosis of breast cancer – comparison of T1 and T2

**Time**	**LLIG**<br>**n [a] (%)**<br>(n=288)	**IG**<br>**n [a] (%)**<br>(n=216)	**Total**<br>**n [a] (%)**<br>(n=504)
**Total dietary supplements**			
T1	167 (58.0)	127 (58.8)	294 (58.3)
T2	166 (57.6)	126 (58.3)	292 (57.9)
**Any vitamin**			
T1	139 (48.3)	103 (47.7)	242 (48.0)
T2	134 (46.5)	101 (46.8)	235 (46.6)
**Any mineral/trace mineral**			
T1	130 (45.1)	105 (48.6)	235 (46.6)
T2	113 (39.2)	104 (48.1)	217 (43.1)
**Any combination V/M and/or other dietary supplements**			
T1	120 (41.7)	93 (43.1)	213 (42.3)
T2	118 (41.0)	91 (42.1)	209 (41.5)

[a] Defined to participants who filled out a questionnaire on dietary intake of supplements at both T1 and T2
IG, intervention group; LLIG, low-level intervention group; n, number of participants with available data from T1 and T2; T1, intake of dietary supplements at the end of lifestyle intervention; T2, repeated answer of intake of dietary supplements 24 months after end of lifestyle intervention.

References

1. Deutsche Gesellschaft für Ernährung, Österreichische Gesellschaft für Ernährung, Schweizerische Gesellschaft für Ernährung (2020). Referenzwerte für die Nährstoffzufuhr, Bonn, 2. Auflage, 6. aktualisierte Ausgabe.
2. European Food and Safety Administration (EFSA). Overview on Tolerable Upper Intake Levels as derived by the Scientific Committee on Food (SCF) and the EFSA Panel on Dietetic Products, Nutrition and Allergies (NDA). 2018. Accessed 08.08.2020
3. National Academies Available from: https://nap.nationalacademies.org/search/?rpp=20&ft=1&term=tolerable+upper+intake. Accessed 06.04.2023
